# Dynamic Phosphoproteomic Profiling Identifies Casein Kinase 2 as a Critical Survival Kinase in Quiescent Breast Cancer Cells and a Potential Therapeutic Target for Minimal Residual Disease

**DOI:** 10.3390/cancers18091449

**Published:** 2026-04-30

**Authors:** Lucia Csergeová, Radoslav Janoštiak

**Affiliations:** BIOCEV-First Faculty of Medicine, Charles University, 121 08 Prague, Czech Republic

**Keywords:** phosphoproteomic, CK2, breast cancer, quiescence, stress response

## Abstract

Background: Quiescent cancer cells (QCCs) evade conventional therapies and contribute to minimal residual disease (MRD) and relapse, yet the signaling pathways governing their survival remain poorly understood. Methods: Here, we performed integrative proteomic and phosphoproteomic profiling of triple-negative breast cancer (TNBC) cells transitioning between proliferation and serum removal-induced quiescence, followed by re-stimulation. Results: We identified dynamic remodeling of both proteome and phosphoproteome, with quiescent cells showing downregulation of mitotic drivers and upregulation of extracellular matrix components. Notably, phosphorylation of CK2 substrates was increased during quiescence, and CK2 inhibition using CX-4945 impaired cell survival under nutrient and genotoxic stress, disrupted autophagy, microtubule dynamics, and protein synthesis. Phospho-enrichment and functional assays identified death-associated protein kinase 3 (DAPK3) as a CK2-regulated effector mediating stress-induced apoptosis. In silico analysis confirms a link between high CK2 expression and poor chemotherapy response in basal breast cancer. Conclusions: These findings establish CK2 as a critical survival kinase in QCCs and a potential therapeutic target for MRD eradication in breast cancer.

## Simple Summary

Some cancer cells can enter a dormant, non-dividing state known as quiescence, which allows them to survive treatment and later drive disease recurrence. These quiescent cancer cells are difficult to target because most therapies are designed to eliminate rapidly dividing cells. In this study, we used advanced protein and phosphorylation profiling to investigate how breast cancer cells adapt to and survive in a quiescent state. We identified the enzyme Casein Kinase 2 (CK2) as a key factor that helps these cells withstand stress conditions such as lack of nutrients or chemotherapy. Blocking CK2 reduced cancer cell survival and increased their sensitivity to treatment. We also found that CK2 may act by suppressing a cell death pathway involving the protein DAPK3. These findings suggest that targeting CK2 could help eliminate therapy-resistant cancer cells and improve long-term treatment outcomes in aggressive breast cancers.

## 1. Introduction

One of the hallmarks of cancer cells is their high proliferative capacity, which has been the primary target of conventional anticancer therapies such as chemotherapy and radiation [1,2,3]. These therapies are highly effective against fast proliferative cells; however, they fail to kill slow-cycling or non-cycling cells—so-called quiescent cancer cells (QCCs) [4,5]. It has been shown that within the tumor microenvironment there is a stable subpopulation of QCCs which is dynamically regulated and modulated by environmental factors such as hypoxia, nutrient deprivation, loss of cell adhesion, and exposure to anticancer treatments [6,7,8]. They have been identified across various tumor types, including glioblastoma, lung, breast and colorectal cancers [9,10,11,12]. Even though this population represents only a fraction of the total cell number (as low as 0.5%) [11], it has significant implication for anticancer therapy efficacy as it can persist through the treatment and give rise to minimal residual disease [9,11,13]. Importantly, QCC can survive long periods of time (years) and lead to disease recurrence which is observed in up to 85% of ovarian cancer patients, 30% of breast, 40% of prostate, and nearly 100% of glioblastoma cancer cases [14].

In mammalian cells, the induction and maintenance of quiescence are regulated by a complex interplay of extrinsic and intrinsic signals. Ultimately, these signals converge on the regulation of cyclin-dependent kinases (CDKs), which determine whether a cell re-enters the cell cycle or remains quiescent. Quiescence is promoted by the upregulation of CDK inhibitors like p27 and p21, downregulation of CDKs, and hypo-phosphorylation of the retinoblastoma (Rb) protein. For instance, during serum starvation, increased expression of p27 suppresses CDK4/6 activity, maintaining cells in the G0 phase [15]. Quiescent cells are typically identified by high p27 and low Ki67 expression [16,17], and may also exhibit specific signaling states such as ERK1/2^high^/p38^low^, or express transcriptional repressors like NR2F1 and kinases such as DYRK1A and DYRK1B [18,19,20].

Given the capacity of QCC to persist in a cell cycle arrested state and later reignite tumor growth, QCCs pose a significant clinical challenge. Effective strategies to target these cells could involve (i) enhancing initial treatment efficacy through improved fractional killing, (ii) permanently locking them out of the cell cycle (e.g., inducing senescence), and (iii) developing therapies that selectively eliminate them. Importantly, quiescence is distinct from senescence—the latter being a permanent cell cycle exit state—making the potential for re-stimulation and relapse unique to quiescent cells [5].

Since quiescence is also a survival mechanism, it can be induced by stress response pathways [21]. In this context, Casein Kinase 2 (CK2) has emerged as a key player in regulating cellular stress responses and survival pathways. CK2 is a constitutively active serine/threonine kinase involved in cell cycle control, apoptosis, transcription, and DNA repair [22,23]. It modulates a wide range of signaling proteins, including p53, NF-κB, and components of the unfolded protein response. By stabilizing antioxidant transcription factors like NRF2 and maintaining the function of stress-protective proteins such as heat shock proteins, CK2 enhances cell survival under adverse conditions [24,25]. However, its sustained activation in tumors may support the survival of quiescent or therapy-resistant cells, contributing to disease persistence and recurrence [26,27]. As such, CK2 represents a potential therapeutic target, particularly in strategies aimed at disrupting tumor dormancy and eliminating minimal residual disease.

To characterize the signaling events on the transition between proliferation and quiescence of cancer cells, we conducted an unbiased phosphoproteomic screen. We analyzed a set of conditions, including continuously proliferating cells, cells after 48 and 96 h of serum removal and cells after serum re-stimulation after 20 min and 120 min, and conducted unbiased proteomic and phosphoproteomic screening to identify novel signaling hubs regulating the transition. Our screen identified CK2 as an important kinase regulating survival of quiescent cells and shows that inhibition of CK2 impairs survival of cells upon nutrient and genotoxic stress. Moreover, we show that death-associated kinase 3 lies downstream of CK2 and is partially responsible for mediating the effect of CK2 inhibition.

In conclusion, through an unbiased phosphoproteomic screen, we identified CK2 as an important survival kinase in breast cancer and targeting CK2 could be a therapeutic option for minimal residual disease (MRD) eradication.

## 2. Materials and Methods

### 2.1. Cell Lines and Cultivation

MDA-MB-231, Hs578T and BT-549 were obtained from American Type Culture Collection (ATCC; Manassas, VA, USA). These cell lines were selected to represent triple-negative breast cancer (TNBC), are enriched in quiescent cancer cell populations and are strongly associated with minimal residual disease (MRD) and therapy resistance, making them a relevant model for studying quiescence-associated survival mechanisms. These cell lines represent genetically and phenotypically distinct TNBC models, allowing us to capture inter-tumoral heterogeneity and improve robustness of our findings. MDA-MB-231 cells are widely used as a mesenchymal-like, highly invasive TNBC model, while Hs578T and BT-549 provide complementary models.

MDA-MB-231 and Hs578T cell lines were cultivated in DMEM—high glucose (Sigma-Aldrich, St. Louis, MO, USA). The BT-549 cell line was cultivated in RPMI (Sigma-Aldrich) reflecting standard conditions established for these cell lines. Cells were cultured in media recommended for each cell line to ensure optimal growth conditions and physiological relevance. Culture media were supplemented with 10% fetal bovine serum (FBS, Biosera, Nuaille, France), penicillin-streptomycin (100 U/mL, Sigma-Aldrich). All cell lines were routinely screened for mycoplasma contamination.

### 2.2. Experimental Design and Sample Collection

Cells were subjected to a defined time-course experimental design to capture transitions between proliferation, quiescence, and reactivation. After initial plating, asynchronously proliferating cells were collected as proliferative control. Cells were then serum-starved for 48 h followed by serum re-stimulation for 24 h to synchronize the population. Quiescence was subsequently induced by serum removal for 48 and 96 h. To assess re-entry into the cell cycle, the serum was reintroduced and samples were collected after 20 and 120 min. All samples were collected in pentaplicates and processed simultaneously for proteomic and phosphoproteomic analysis.

### 2.3. Cell Viability Assay

Cell viability was assessed using the MTT assay as previously described [28,29]. MDA-MB-231, BT-549 cells (10,000 cells/well) and Hs578T cells (4000 cells/well) were seeded into 96-well plates and cultured in the appropriate media type (DMEM/ RPMI) containing either 10% fetal bovine serum (FBS) or serum-free media. Cells were treated with respective compounds (CK2 inhibitor—CX-4645, Monmouth Junction, NJ, USA, Doxorubicin, Sigma-Aldrich, St. Louis, MO, USA) and incubated at 37 °C in a 5% CO_2_ atmosphere for indicated timepoints dependent on the assay. At the designated timepoints, 20 µL of MTT solution (5 mg/mL) was directly added to each well containing 200 µL of media. The cells were then incubated for 1 h at 37 °C. Metabolically active cells reduce the yellow tetrazolium salt MTT to insoluble purple formazan crystals via mitochondrial dehydrogenase activity. Following incubation, the MTT solution was removed, and the formazan crystals were solubilized by adding 100 µL of dimethyl sulfoxide (DMSO) to each well and the absorbance was measured at 595 nm using a Tecan Infinite^®^ M200 Pro microplate reader (Tecan, Männedorf, Switzerland). The measured absorbance is directly proportional to the number of metabolically active (viable) cells and thus serves as an indirect indicator of cell viability and proliferation.

### 2.4. Colony Formation Assay

For the colony formation assay, previously published protocol described in Franken et al. was followed [30]. Briefly, 10,000 cells (MDA-MB-231, BT-549, Hs578T) per well were seeded into 6-well plates and cultured in media supplemented with 10% fetal bovine serum (FBS). Cells were treated with either DMSO (vehicle control) or increasing concentrations of the CK2 inhibitor (CX-4945): 0.2 µM, 0.5 µM, 1 µM, 2 µM, and 5 µM. Afterwards, cells were incubated at 37 °C in a humidified atmosphere with 5% CO_2_ for 10 days. Following the incubation, the culture media was removed, and the wells were washed twice with phosphate-buffered saline (PBS). Colonies were then fixed and stained with Crystal Violet Solution (5 mg/mL crystal violet in 10% ethanol, 50% methanol, and 40% water). After staining, excess dye was removed by rinsing with water. Images of the stained colonies were captured using a ChemiDoc™ MP Imaging System (Bio-Rad, Hercules, CA, USA). Quantification was carried out using ImageJ software [31] (version 1.46r).

### 2.5. Immunoblotting

Cell cultures were washed with phosphate-buffered saline (PBS) and lysed in RIPA buffer (150 mM NaCl; 50 mM Tris-HCl, pH 7.5; 0.5% DOC; 0.1% SDS; 1% Triton) supplemented with 1× Halt^TM^ Protease and Phosphatase inhibitor cocktail (Thermo Fisher Scientific, Waltham, MA, USA, Cat# 78446). Protein concentrations in lysates were determined using the Pierce^TM^ BCA Protein Assay (Thermo Fisher Scientific, Cat# A65453). Protein lysates were diluted in Laemmli sample buffer (4× concentrated: 0.5 M Tris-HCl, pH 6.8; 20% SDS; 40% glycerol; 0.02% Bromophenol blue; 0.2 M DTT).

For immunoblotting, samples were separated on SDS-polyacrylamide gels and transferred onto PVDF membranes. Non-specific activity was blocked by incubating membranes for 60 min at room temperature in Tris-buffered saline (TBS) with 0.1% Tween^®^ 20 detergent (TBST) containing 5% non-fat dry milk. After protein transfer, membranes were horizontally cut into sections corresponding to the molecular weight range of the target proteins (typically ±20–30 kDa around the expected size). Each membrane segment was subsequently processed independently for antibody incubation. Membranes were then incubated overnight at 4 °C with primary antibodies, washed 3 times with TBST, and incubated for 1 h at room temperature with horseradish peroxidase (HRP)—conjugated secondary antibodies. After washing for 4 times in TBST, the blots were developed using the Immobilon^®^ ECL Ultra Western HRP Substrate (Millipore, Burlington, MA, USA, Cat# WBULS0100) and ChemiDoc^TM^ MP Imaging system (Bio-Rad, Hercules, CA, USA). Quantification of Western blots was carried out using ImageJ software (https://imagej.net/ij/, accessed on 10 May 2025). Antibodies used in the study are listed in Table S1.

### 2.6. De Novo Proteosynthesis Assay

De novo proteosynthesis was assessed using the Click-iT^TM^ HPG Alexa Fluor^TM^ 488 Protein Synthesis Assay Kit (Invitrogen, Carlsbad, CA, USA, Cat# C10428). Cells were pre-treated with 1 µM, 5 µM, or without CK2i for 2 days before the assay followed by incubation with 50 µM Click-iT^TM^ HPG for 2 h, trypsinized and washed twice with PBS. De novo proteosynthesis was assessed following the manufacturer’s protocol. In brief, cells were fixed using 3.7% formaldehyde for 15 min at room temperature, followed by two washes with 200 µL of 3% BSA in PBS. Permeabilization was performed by incubating cells with 200 µL of 0.5% Triton^®^ X-100 in PBS for 20 min at room temperature, followed by two additional washes with 3% BSA in PBS. Next, 200 µL of the Click-iT^TM^ reaction cocktail (containing 5 times less Alexa Fluor^TM^ azide) was added, and cells were incubated accordingly. After incubation, they were washed with 200 µL of Click-iT^TM^ reaction rinse buffer and resuspended in PBS. Protein synthesis was analyzed using the CytoFLEX flow cytometer (Beckman Coulter, Life Sciences, Brea, CA, USA) and data were processed using FloJo software, version 10.10.0 (BD).

### 2.7. Immunoprecipitation

For immunoprecipitation experiments, cells were grown on a 10 cm dish and treated with CX-4945 or vehicle for 24 h. Cells were lysed in immunoprecipitation buffer (10 mM Tris (pH 7.5), 1% NP-40, and 2 mM EDTA) supplemented with 1x Halt^TM^ Protease and Phosphatase inhibitor cocktail (Thermo Fisher Scientific, Cat# 78446) and protein concentration was estimated using the Pierce^TM^ BCA Protein Assay (Thermo Fisher Scientific, Cat# A65453). Protein A/G magnetic beads (Pierce^TM^, Thermo Fisher Scientific, Waltham, MA, USA) were washed in immunoprecipitation buffer and incubated with target antibody or isotype control (1 μg) for 2 h at 4 °C with gentle rotation. Afterwards, beads were washed to remove unbound antibodies and mixed with equal amounts of protein lysates (1000 μg) and incubated overnight at 4 °C with gentle rotation. After incubation, beads were washed 4 times in immunoprecipitation buffer and subsequently analyzed using Western blot or mass spectrometry. The immunoprecipitation protocol followed previously published work [32], modified as described. For Western blot analysis, immunoprecipitated proteins were eluted by incubating the beads in Laemmli sample buffer followed by boiling at 95 °C for 5 min. Samples were then resolved by SDS-PAGE and analyzed by immunoblotting. For mass spectrometry analysis, beads were processed for on-bead digestion as described in Section 2.11.1. Briefly, proteins bound to beads were reduced and alkylated, followed by digestion with trypsin overnight at 37 °C. Peptides were subsequently collected, desalted using C18 stage tips, and subjected to LC-MS/MS analysis as described below.

### 2.8. Cell Cycle Analysis

Indicated cell lines were treated with CX-4549 at the indicated concentration for 24 h, harvested by trypsinization, washed twice with ice-cold PBS, and fixed dropwise in 70% ethanol while vortexing gently for 24 h at −20 °C. Prior to staining, cells were washed with PBS and incubated in staining buffer containing 50 µg/mL propidium iodide (PI), 100 µg/mL RNase A, and 0.1% Triton X-100 in PBS for 30 min at room temperature in the dark. Stained cells were analyzed using the CytoFLEX flow cytometer (Beckman Coulter Life Sciences) and data were processed using FlowJo software (BD). Cell cycle distribution was determined by quantifying the percentage of cells in G0/G1, S, and G2/M phases based on DNA content.

### 2.9. LysoTracker Staining

Cells were treated with indicated concentrations of CX-4549 for 24 h, trypsinized, counted, and 500,000 cells were stained with 50 nM Lysotracker^TM^ Green DND-26 (Thermo Fisher Scientific) for 5 min. After incubation, cells were washed two times and resuspended in PBS + 1 m EDTA + 1% BSA. Finally, cells were analyzed using the CytoFLEX flow cytometer (Beckman Coulter Life Sciences) and mean fluorescence intensity was extracted using FlowJo software (BD).

### 2.10. Microtubule Regrowth Assay

Indicated cell lines were plated onto coverslips and cultivated overnight at 37 °C in a humidified incubator. Cells were pretreated with the indicated concentration of CK2i for 2 h followed by treatment with 10 µM nocodazole (Merck, Darmstadt, Germany) for 3 h at 37 °C to depolymerize microtubules. After treatment, cells were washed three times with warm PBS to remove residual nocodazole and immediately transferred to a pre-warmed nocodazole-free complete medium to allow microtubule regrowth. Regrowth was allowed to proceed at 37 °C for 15 min and cells were fixed with 4% paraformaldehyde in PBS for 10 min at room temperature followed by permeabilization with 0.25% Triton X-100 in PBS for 10 min. Cells were then blocked with 5% BSA in PBS for 30 min, followed by incubation with anti-α-tubulin antibody (DM1A, 1:200, SantaCruz Biotechnology, Dallas, TX, USA) for 2 h at room temperature. After washing, coverslips were incubated with Alexa Fluor 488-conjugated anti-mouse secondary antibody (1:1000) for 1 h at room temperature. Nuclei were counterstained with DAPI, and coverslips were mounted using ProLong^TM^ Mountant (Invitrogen). Images were acquired using the Leica Dmi8 inverted fluorescence microscope (Leica, Wetzlar, Germany).

### 2.11. Mass Spectrometry

#### 2.11.1. Protein Digestion

Individual samples were mixed with 2% SDC in 100 mM TRIS buffer (pH 8.5), boiled at 95 °C for 5 min and further sonicated using a micro probe sonicator (Bandelin Sonoplus, BANDELIN electronic GmbH & Co. KG, Berlin, Germany). Protein concentration was determined using the BCA protein assay kit (ThermoFisher Scientific).

Protein digestion workflow followed established protocol as described [33]. Briefly, 250 µg of protein per sample was reduced with 10 mM TCEP and alkylated with 40 mM chloroacetamide (CAA). The pH of the solution was adjusted to approximately 8.5 using 5 M KOH to ensure optimal conditions for trypsin activity. Samples were incubated at 45 °C for 5 min and subsequently digested with sequencing-grade trypsin (1:50 enzyme-to-protein ratio) overnight at 37 °C. Phosphopeptides were enriched using TiO_2_ according to Humphrey et al. [33]. After enrichment, peptides were desalted using in-house made stage tips packed with C18 disks (Empore) according to Rappsilber et al. [34].

#### 2.11.2. nLC-MS 2 Analysis

Nano-reversed phase columns (Ion Opticks Ultimate (Collingwood, Australia) TS 25 cm × 75 µm ID, C18 UHPLC column, 1.7 µm particles, 120 Å pore size) were used for LC/MS analysis. Mobile phase buffer A was composed of water and 0.1% formic acid. Mobile phase B was composed of acetonitrile and 0.1% formic acid. Samples (500 ng per sample) were individually loaded onto the trap column (C18 PepMap100, 5 μm particle size, 300 μm × 5 mm, Thermo Scientific) for 1 min at 18 μL/min. The loading buffer was composed of water, 2% acetonitrile and 0.1% trifluoroacetic acid. Peptides were eluted with Mobile phase B gradient from 4% to 25% B in 28 min and from 25% B to 35% in the next 2 min followed by a 5 min wash with 75% B. Eluting peptide cations were converted to gas-phase ions by electrospray ionization and analyzed on a Thermo Orbitrap Ascend (Thermofisher Scientific, Bremen, Germany) individually resulting in 25 separate LC-MS/MS runs. Survey scans of peptide precursors from 350 to 1400 *m*/*z* were performed in orbitrap at 120 K resolution (at 200 *m*/*z*) with a 100% ion count target. Tandem MS was performed by isolation at 1.6 Da with the quadrupole, and CID fragmentation was performed with normalized collision energy of 30% and 10 ms activation time. Fragmentation spectra were acquired in ion trap with the scan rate set to Normal. The MS2 ion count target was set to 200% and the max injection time was 200 ms. Only those precursors with charge state 2–6 were sampled for MS2. The dynamic exclusion duration was set to 30 s with a 10 ppm tolerance around the selected precursor and its isotopes. Monoisotopic precursor selection was turned on. Cycle time was set to 2 s.

#### 2.11.3. Data Analysis

All data were analyzed and quantified with the MaxQuant software (version 2.4.13.0) [35]. The false discovery rate (FDR) was set to 1% for both proteins and peptides and we specified a minimum peptide length of seven amino acids. The Andromeda search engine was used for the MS/MS spectra search against the Human database (downloaded from Uniprot in March 2023, *Homo sapiens*, containing 20,605 entries). Enzyme specificity was set as C-terminal to Arg and Lys, also allowing cleavage at proline bonds and a maximum of two missed cleavages. Carbamidomethylation of cysteine was selected as the fixed modification and N-terminal protein acetylation, methionine oxidation and serine, threonine and tyrosine phosphorylation as variable modifications. The “match between runs” feature of MaxQuant was used to transfer identifications to other LC-MS/MS runs based on their masses and retention time (maximum deviation 0.7 min), and this was also used in quantification experiments. Quantifications were performed with the label-free algorithm in MaxQuant [36]. MBR is controlled within the MaxQuant framework and maintains the overall FDR threshold at 1%. All experimental conditions were analyzed in pentaplicates, resulting in a total of 25 LC-MS/MS runs. Data analysis was performed using Perseus 1.6.15.0 software [37]. Only those phosphosites with localization probability higher than 0.75 were used for further data analysis.

### 2.12. Dataset Analysis

Publicly available proteomic and genomic data were obtained from the Clinical Proteomic Tumor Analysis Consortium (CPTAC) breast cancer cohort via the cBioPortal platform (https://www.cbioportal.org, accessed on 10 May 2025). The dataset included 122 breast cancer patient samples with matched proteomic and genomic profiling. Proteomic and phosphoproteomic data were obtained as relative expression and phosphorylation values from the CPTAC breast cancer dataset via cBioPortal. The provided normalized values were used directly for downstream analyses. Protein and phosphosite abundance levels were compared between MYC-amplified and non-amplified groups. Patients were stratified based on MYC copy number alteration status using GISTIC-derived calls provided within cBioPortal. Samples classified as “amplified” were assigned to the MYC-amplified group, while all other samples were included in the non-amplified group. Kaplan–Meier survival analyses were performed using the KMplot online platform (http://kmplot.com/analysis/, accessed on 10 May 2025), which integrates gene expression and clinical data from publicly available breast cancer cohorts. For this study, the breast cancer mRNA gene chip dataset comprising 4929 patients was used [38]. Patients were stratified into high- and low-expression groups based on the median expression of CSNK2A1. Group sizes were automatically determined by the platform following median-based stratification, resulting in approximately equal cohort sizes. Survival differences between groups were evaluated using the log-rank (Mantel–Cox) test as implemented within the platform. Hazard ratios (HR) with 95% confidence intervals and corresponding *p*-values were obtained directly from the tool. Analyses were performed using default settings unless otherwise specified. The dataset and analytical approach are described in detail in previous publications [38].

### 2.13. Data Visualizations

Heatmaps, volcano plots, and Gene Ontology (GO) pathway analyses were performed using SRplot [39], an interactive web-based visualization platform for omics data. Kaplan–Meier curves were constructed using the kmplot webtool platform [40]. Kinase motif logos were generated using the PhosphoSitePlus web platform [41].

### 2.14. Statistical Analysis

All quantitative data are presented as mean ± standard deviation (SD) from at least three independent experiments, unless stated otherwise. For comparisons between two groups, an unpaired two-tailed Student’s *t*-test was used. A *p*-value of less than 0.05 was considered statistically significant. Statistical significance is indicated as follows: ns (not significant), *p* < 0.05 (*), *p* < 0.01 (**), *p* < 0.001 (***), and *p* < 0.0001 (****). For proteomic and phosphoproteomic analyses, statistical significance was determined using Student’s *t*-test with a false discovery rate (FDR) threshold of 1%, as implemented in Perseus software (v 1.6.15.0). Kaplan–Meier survival analyses were performed using the KMplot online platform. Survival differences between groups were evaluated using the log-rank (Mantel–Cox) test as implemented in the platform. Hazard ratios (HR) with 95% confidence intervals and corresponding *p*-values were obtained directly from the tool.

## 3. Results

### 3.1. Phosphoproteomic Analysis of Serum Removal-Mediated Quiescence Induction and Re-Entry into Proliferation

**Figure 1:**
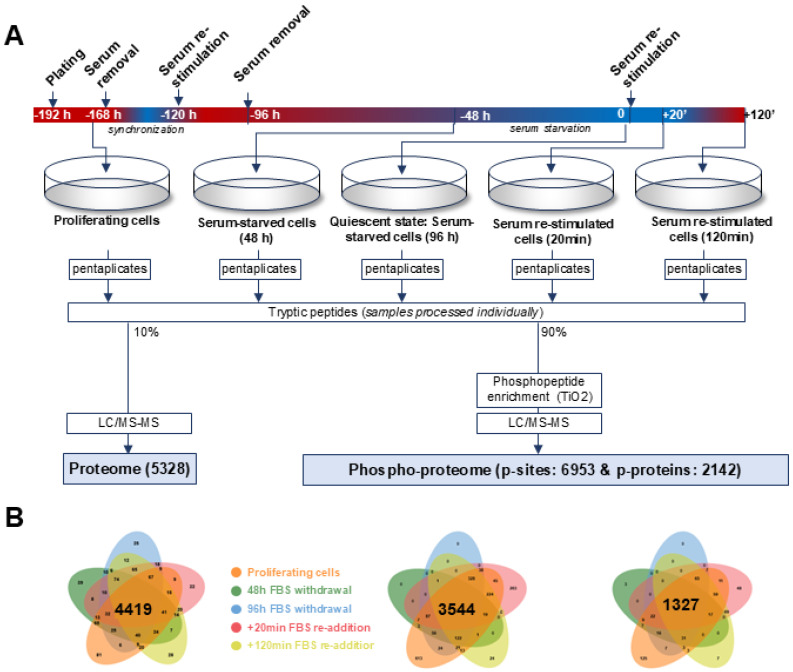
Experimental design for proteomic and phosphoproteomic profiling of quiescence entry and exit. (**A**) Schematic of the experimental workflow showing timepoints for sample collection: continuously proliferating cells (proliferative), serum-starved cells (48 h and 96 h (quiescent cells)), and serum re-stimulated cells (+20 min and +120 min). Cells were processed in pentaplicates for label-free mass spectrometry following tryptic digestion and phosphopeptide enrichment. Proteome coverage included 5328 proteins and 6953 phosphosites across all timepoints. (**B**) Ven diagrams indicating numbers of identified peptides (left), phosphosites (middle) and phospho-peptides (right) in each condition.

To date, relatively few large-scale proteomic and phosphoproteomic studies have systematically compared proliferating and arrested cells. Many of these have been conducted in non-transformed systems or have focused on steady-state conditions rather than dynamic transitions between proliferation and quiescence [36,42,43,44,45,46]. To address the gap, we conducted performed and unbiased proteomic and phosphoproteomic analysis of cells on the transition from proliferation to serum removal-mediated quiescence followed by serum addition-mediated re-entry in the cell cycle (Figure 1). For the analysis we selected TNBC cell line MDA-MB-231 as a model based on several reasons. It represents the most aggressive breast cancer tumor subtype with 5-year survival at 77% for all stages, and 12% for stage IV disease. This subtype is associated with higher rates of metastasis formation and tumor recurrence peaking at 2–3 years, indicating presence of QCCs [14,47]. Additionally, QCC population seems to be enriched in TNBC to a bigger extent in comparison to other breast cancer subtypes [48]. To study the transition, we enriched the QCC population by serum removal as a quiescence inductor. This represents a more physiological condition compared to cytotoxic compounds or pharmacological induction (e.g., using CDK4/6 inhibitors) [49]. Workflow and sample collection timepoints are outlined in Figure 1A. To validate the approach and selected timepoints, we analyzed the status of the cells using Western blot for key proteins indicating proliferative status—Rb1 phosphorylation, p27Kip and E2F8. Hypophoshorylation of Rb1, absence of E2F8 and increased levels of p27Kip are well established markers of cells in the quiescent state [50,51,52]. Our Western blot analysis shows hyperphosphorylated Rb1, high levels of E2F8 and low levels of p27Kip in continuously proliferating cells (Figure S1). On the other hand, levels of hyper-phosphorylated Rb1 and total E2F8 are low in 96 h serum-starved cells, while the level of p27Kip is the highest, indicating significant enrichment of quiescent cells population (Figure S1). After validation of the approach, the samples for unbiased phosphoproteomic analysis were collected in pentaplicates and processed at the same time, followed by label-free mass spectrometry analysis. Overall, the proteomic analysis identified 5328 individual proteins across all the conditions, out of which 4419 proteins were identified in all the conditions in every replicate (Figure 1B, left). Furthermore, phosphoproteomic analysis identified 6953 phosphosites present on 2142 proteins across all the conditions. Phosphosites identified across all the conditions were significantly reduced by almost 50% in both phosphosites (3544 sites identified in all conditions) as well as phosphorylated proteins (1327 sites identified in all conditions). These changes were most likely attributable to biological changes in global phosphorylation dynamics associated with quiescence, rather than technical limitations. Entry into quiescence is accompanied by a widespread decrease in kinase-driven signaling, particularly involving cyclin-dependent kinases and mitotic regulators, resulting in reduced phosphorylation of proteins involved in cell cycle progression, transcription, and translation. Consistent with this, we observed substantially higher numbers of identified phosphosites in continuously proliferating cells compared to serum-starved conditions. This increase was observed despite identical sample preparation and mass spectrometry analysis, supporting a biological rather than technical origin of this effect (Figure 1B, right).

### 3.2. Global Cellular Proteome Is Significantly Remodeled at the Exit of Cells from the Quiescent State

**Figure 2:**
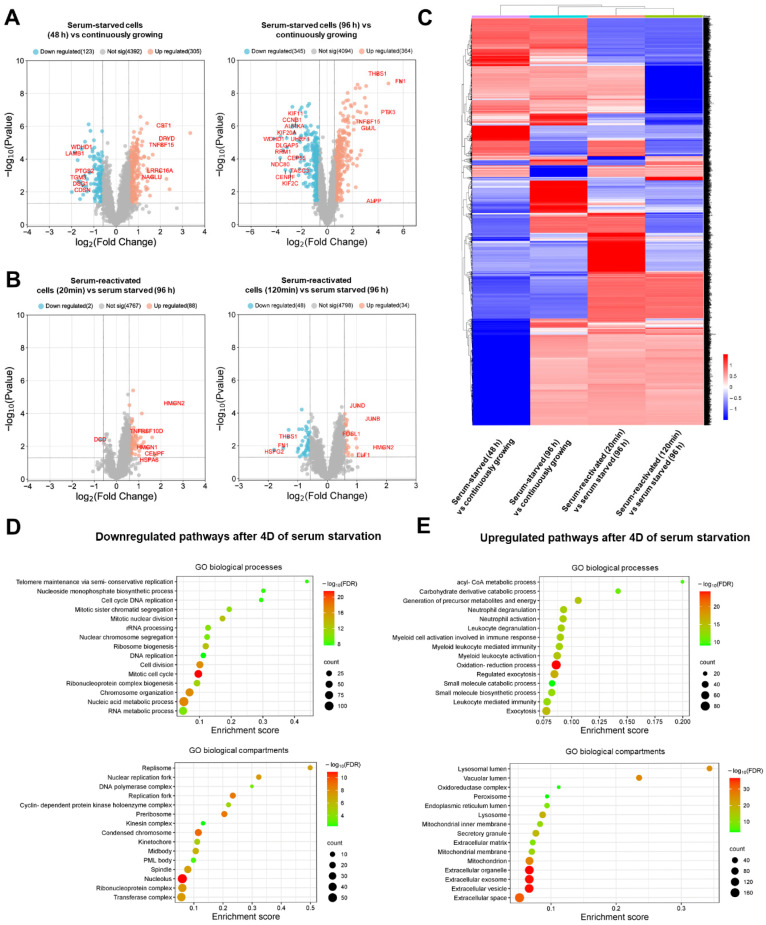
Global proteome remodeling during quiescence and re-stimulation. (**A**) Volcano plots comparing protein expression at defined timepoints (left: 48 h; right: 96 h) relative to continuously proliferating cells. Dashed lines indicate thresholds of (log2FC) ≥ 0.58 (1.5-fold change) and *p* < 0.05. (**B**) Volcano plots comparing protein expression at defined timepoints (left: 48 h; right: 96 h) relative to serum-starved states. Dashed lines indicate thresholds of (log2FC) ≥ 0.58 (1.5-fold change) and *p* < 0.05. (**C**) Heatmap of differentially expressed proteins across the indicated conditions. Values are represented as z-score normalized log2-transformed intensities. Red indicates higher relative expression, while blue indicates lower relative expression across the dataset. (**D**) GO enrichment analyses (top: GO biological processes; bottom: GO biological compartments) showing downregulated pathways after 4D (96 h) of serum starvation compared to continuously proliferating cells. Color indicates false discovery rate (FDR), circle size indicates number of identified hits in the pathway, X-axis shows enrichment score. (**E**) GO enrichment analyses (top: GO biological processes; bottom: GO biological compartments) showing upregulated pathways after 4D (96 h) of serum starvation compared to continuously proliferating cells. Color indicates false discovery rate (FDR), circle size indicates number of identified hits in the pathway, X-axis shows enrichment score.

To interpret the complex patterns emerging from mass spectrometry (MS)-based proteomics, we first constructed volcano plots identifying key changes in cells exiting proliferation and entering quiescence (Figure 2A). While we see significant changes (defined as ≥1.5-fold change (log2FC ≥ 0.58, *p*-value ≤ 0.05)) in proteome after 48 h of serum removal, these changes are much more pronounced in cells after 96 h of serum removal. A threshold of ≥1.5-fold change was selected to balance statistical significance with biological relevance, as moderate fold changes can reflect meaningful regulatory events in signaling pathways, particularly in phosphoproteomic datasets [33,36,42,45]. As expected, we can see significant downregulation of key proliferation drivers such as CCNB1, DLGAP5, AURKA, KIF11 or TACC3 and concomitant upregulation of tumor microenvironment-affecting proteins such as FN1, THBS1, TNFSF15 and PTX3 (Figure 2A). On the other hand, proteome changes in cells at the exit from quiescence into proliferation mode are less pronounced. However, it uncovers interesting signaling events governing the first steps in the process. At the 20 min timepoint after serum re-stimulation, we could see only minor global changes, as expected. Interestingly, we identified HMGN2 as a key protein that is being translated, which is in line with its role as a chromatin remodeler enabling expression of key cell cycle genes [53] (Figure 2B, left). At the 120 min timepoint after serum re-activation, we can see stimulation of the expression of key transcription factors driving the cell cycle—Jun B, Jun D, FOSL1 and ELF1. On the other hand, we can already see degradation of quiescence-maintaining proteins such FN1, THBS1 and HSPG2, indicating a key role of proteasome degradation in the quiescence exit (Figure 2B, right). To further explore global proteome dynamics, we constructed a heatmap of differentially expressed proteins across all conditions (Figure 2C). The heatmap reveals a gradual and coordinated remodeling of the proteome during the transition from proliferation to quiescence and subsequent re-stimulation. Distinct clusters of proteins are evident, including those progressively downregulated during quiescence, largely associated with cell cycle progression and mitosis, and those upregulated in quiescent cells, enriched for extracellular matrix and stress-response-related proteins. Upon serum re-stimulation, these patterns are partially reversed, reflecting early stages of proteome reprogramming during cell cycle re-entry. Together, these data highlight the dynamic and reversible nature of proteome remodeling during quiescence transitions. Finally, we analyzed enriched GO biological processes and compartments specifically in the quiescent condition (Figure 2D,E). As expected, major downregulated pathways are associated with DNA replication, cell cycle progression and mitosis (Figure 2D). On the contrary, pathways such as oxidative phosphorylation or exocytosis are significantly upregulated (Figure 2E). Interestingly, pathways regulating extracellular matrix remodeling are most enriched in quiescent cells, further emphasizing the key role of the quiescence niche (Figure 2E, bottom).

### 3.3. Protein Phosphorylation Network Regulates Cellular Behavior at the Transition Points Between Quiescence and Proliferation

**Figure 3:**
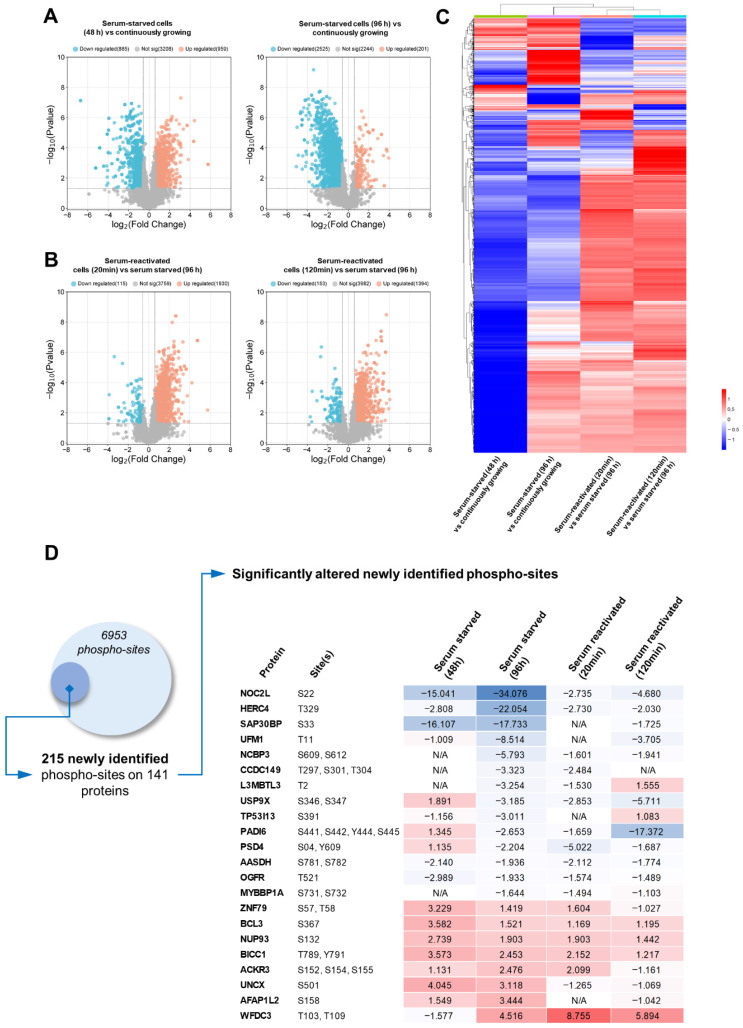
Phosphoproteomic dynamics during quiescence and exit. (**A**) Volcano plots comparing site-specific phosphorylation levels at defined timepoints (left: 48 h; right: 96 h) relative to continuously proliferating cells. Dashed lines indicate thresholds of (log2FC) ≥ 0.58 (1.5-fold change) and *p* < 0.05. (**B**) Volcano plots comparing site-specific phosphorylation levels at defined timepoints (left: 48 h; right: 96 h) relative to serum-starved states. Dashed lines indicate thresholds of (log2FC) ≥ 0.58 (1.5-fold change) and *p* < 0.05. (**C**) Heatmap of differentially phosphorylated sites across the time course. (**D**) Table summarizing novel phosphosites significantly altered during quiescence–proliferation transitions, with selected examples. Values represent fold changes in the abundance of quantified phosphopeptides. For serum-starved conditions (48 h and 96 h), values are calculated relative to continuously proliferating cells. For serum re-stimulation conditions (+20 min and +120 min), values are calculated relative to quiescent (96 h serum-starved) cells. Red indicates increased phosphorylation, blue indicates decreased phosphorylation in our phosphoproteomic dataset.

To complement the data from proteomic analysis, we analyzed global changes in protein phosphorylation at the transition between proliferation and quiescence in pentaplicates. First, we constructed volcano plots (Figure 3A,B) and heatmaps (Figure 3C) to investigate the dynamics of global protein phosphorylation. Both analyses showed a continuum of phosphorylations. A total of 48 h after quiescence induction, we see similar amounts of phosphosites being upregulated, as well as downregulated, indicating active signaling rewiring (Figure 3A, left). In the condition enriched for quiescent cells, we see the majority of the phosphosites being downregulated, confirming lower activity of the signaling pathways (Figure 3A, right). On the other hand, after serum re-stimulation and cell signaling reactivation, we see a significant shift in phosphorylation. The majority of significantly altered phosphosites are upregulated in early (94%) as well as late (90%) quiescence exit stages (Figure 3B). More detailed analysis of altered phosphorylation reveals patterns specific to individual stages. As expected, activating phosphorylation of proteins regulating the cell cycle (Rb1, SRSF1), transcription (CTR9, TCOF1), translation (4EBP1, EIF3D) and mitosis (LMNB2, TOP2A, Ki67, TPX2) was downregulated in both serum-starved conditions (File S1, Figure 3). On the other hand, we identified proteins with significantly increased site-specific phosphorylation in the quiescent stage. SCRIB, EDC4, CIC, SAP30, MAP1B, SP4 and STARD3NL were upregulated by more than 10-fold in comparison to continuously proliferating cells (File S1). Phosphorylation of these proteins could serve as active quiescence-maintaining signaling that might be targeted to eliminate QCCs. While we have identified numerous previously identified sites, we have also identified 215 previously undescribed phosphorylations present on 141 individual proteins (Figure 3D). Out of these novel sites, there are 36 sites whose phosphorylation is significantly altered across different conditions and could represent novel regulatory circuits governing transition between quiescence and proliferation.

### 3.4. Identified Proteomic and Phosphoproteomic Alterations Were Validated In Vitro as Well as in In Silico Patient Datasets

**Figure 4:**
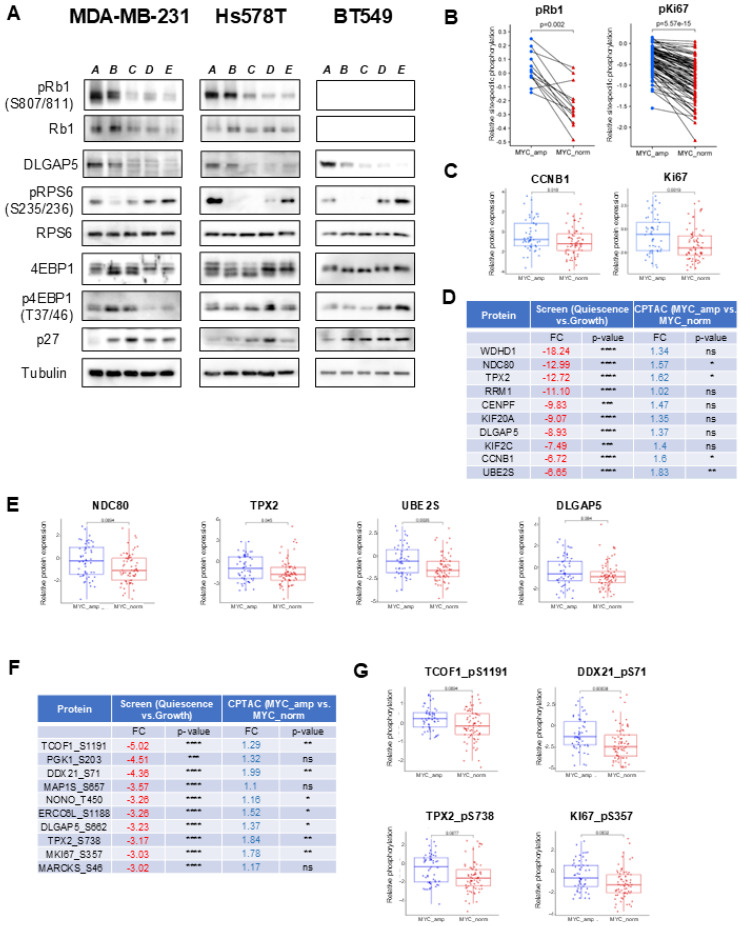
Validation of protein and phosphorylation changes in TNBC cell lines and patient data. (**A**) Expression and site-specific phosphorylation of indicated proteins was analyzed in MDA-MB-231, Hs578T and BT-549 in various timepoints corresponding to the transition between quiescence and proliferation. Timepoints were as follows: *A*—continuously proliferating cells; *B*—serum-starved cells (48 h); *C*—serum-starved cells (96 h); *D*—serum-reactivated cells (+20 min); *E*—serum-reactivated cells (+120 min). α-tubulin was used as loading control. (**B**,**C**) Analysis of site-specific phosphorylation and protein expression of indicated proteins in CPTAC patient samples dataset. (**D**) Comparison table of top quiescence-downregulated proteins in vitro (screening conditions) and in CPTAC patients’ samples dataset. FC—fold change, ns—not significant, *, **, ***, and **** represent *p*-values < 0.05, <0.01, <0.001, and <0.0001. (**E**) Box plots showing relative protein expression levels derived from the CPTAC dataset for proteins significantly altered between MYC-amplified and MYC non-amplified breast tumors. (**F**) Comparison table of top quiescence-downregulated site-specific phosphorylations in vitro (screening conditions) and in CPTAC patients’ samples dataset. FC—fold change, ns—not significant, *, **, ***, and **** represent *p*-values < 0.05, <0.01, <0.001, and <0.0001. (**G**) Box plots showing relative site-specific phosphorylation levels derived from the CPTAC dataset for phosphosites significantly altered between MYC-amplified and MYC non-amplified breast tumors.

Although our proteomic and phosphoproteomic analysis was robust, we followed to validate some of the findings in two sets of experiments using Western blot analysis. First we analyzed total levels as well as site-specific phosphorylation of several proteins in three TNBC cell lines—MDA-MB-231, Hs578T and BT-549. We looked at major cell cycle regulator Retinoblastoma protein 1 and saw that in MDA-MB-231 as well as Hs578T the phosphorylation of CDK-directed sites (S807/S811) was consistently reduced in cells after 96 h of serum starvation and we did not see a significant increase after 20 nor 120 min of serum re-stimulation (Figure 4A). This is consistent with the fact that Rb1 is not hyperphosphorylated until the beginning of the S-phase [54]. Additionally, we analyzed the levels of DLGAP5 as a key mitosis-promoting protein and we could see complete absence of the protein in MDA-MB-231 and Hs578T cells (Figure 4A). However, in Rb1-negative cell line BT-549, DLGAP5 was still present at 96 h after serum starvation. Additionally, p27 as a major quiescence regulatory protein was consistently increased in cells arrested in the G0 phase. However, it was only partially degraded after serum re-stimulation, which is consistent with its role as a platform for CDK4/6-Cycline D complex formation [55]. Finally, our in vitro experiments show major changes in the proteosynthetic pathway, where phosphorylation of RPS6 S235/236 is the most consistent change across all three cell lines (Figure 4A). Interestingly, the total level of 4EPB1 phosphorylation was consistently decreased in serum-starved cells and bounced right back after serum re-stimulation as shown by the total protein bands shifts. However, the exact position of this phosphorylation seems to be cell-line-dependent, as evidenced by a distinct pattern of T37/46 phosphorylation indicating a phosphorylation code rather than site-specific events.

Additionally, we sought to compare our results with publicly available datasets containing samples from patients. To this aim, we analyzed the CPTAC database using cBioPortal webtool (cbioportal.org) comprising 122 patient samples with matched genomic and proteomic data [56]. Patients were stratified into MYC-amplified and non-amplified groups based on GISTIC-derived copy number alteration calls. MYC amplification was used as a surrogate marker of increased proliferative activity, as this genetic alteration has been associated with proliferation-dominant tumor states and correlates with elevated cell cycle progression and higher Ki67 expression across multiple cancer types, including TNBC [56,57]. To validate our stratification approach, we first compared phosphorylation of Rb1 and Ki67 in MYC-amplified (high-proliferation tumors) cohort and MYC non-amplified (low-proliferation tumors) patient cohort. We show that levels of phosphorylation across various sites is significantly decreased for pRb1 (*p* = 0.002) as well as for pKI67 (*p* = 5.57 × 10^−15^) (Figure 4B). Additionally, we analyzed the total level of cell cycle proteins Cyclin B1 and Ki67 and confirmed a higher level of these cell cycle drivers in MYC-amplified breast cancer tumors, supporting the validity of the proliferation-based stratification (Figure 4C).

We then compared protein expression levels of the ten most downregulated proteins identified in our in vitro quiescence model with CPTAC patient data (Figure 4D,E). All proteins showed increased expression in MYC-amplified tumors to varying degrees, with UBE2S, CCNB1, TPX2, and NDC80 reaching statistical significance (*p* < 0.05). These results provide supportive evidence for the relevance of our in vitro proteomic findings. In contrast, comparison of phosphosite-level data revealed limited overlap between datasets (Figure 4F,G). Only a small number of phosphorylation sites, including TCOF_pS1191, DDX21_pS71, TPX2_pS738, and Ki67_S357, were significantly upregulated in the high-proliferation cohort. This limited concordance likely reflects biological differences between controlled in vitro conditions and heterogeneous tumor samples, as well as differences in phosphoproteomic coverage. Therefore, phosphosite-level comparisons should be interpreted as providing partial support rather than direct validation of the in vitro findings.

### 3.5. CK2 Kinase Substrates Are Upregulated in Response to Nutrient Stress

**Figure 5:**
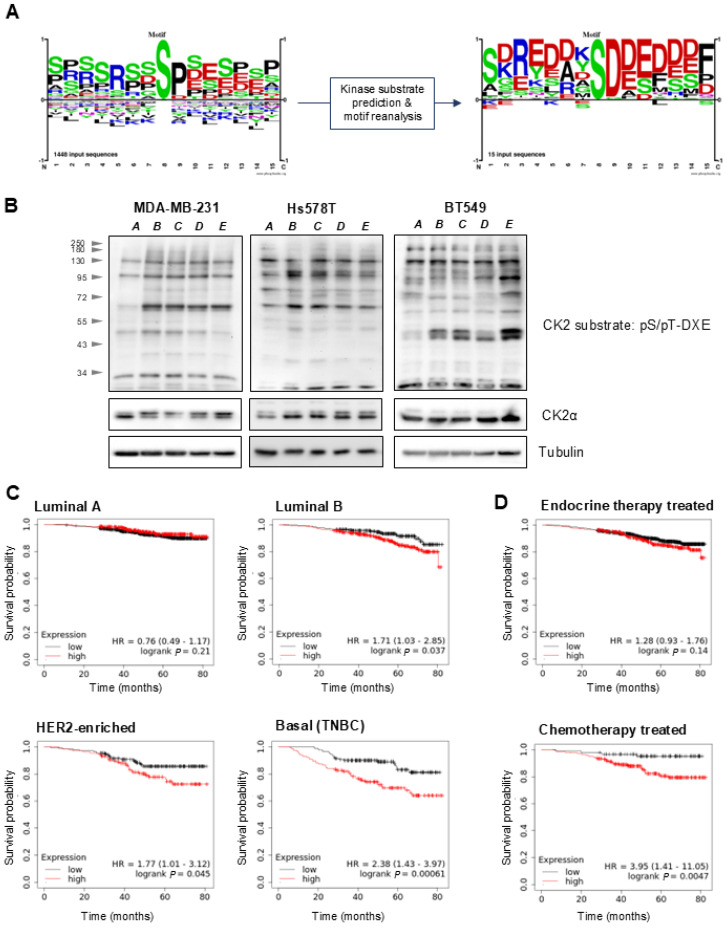
CK2 activity and clinical relevance in breast cancer. (**A**) Kinase motif enrichment identifies CK2 consensus motifs upregulated in quiescent cells. (**B**) CK2-substrate phosphorylation levels in various timepoints corresponding to the transition between quiescence and proliferation was analyzed using Western blot. Timepoints were as follows: *A*—continuously proliferating cells; *B*—serum-starved cells (48 h); *C*—serum-starved cells (96 h); *D*—serum-restimulated cells (+20 min); *E*—serum-restimulated cells (+120 min). α-tubulin was used as loading control. (**C**) Kaplan–Meier curves showing survival probabilities of patients with high and low expression of *CSNK2A1* belonging to PAM50 breast cancer subtypes. HR—hazard ratio. (**D**) Kaplan–Meier curves showing survival probabilities of patients with high and low expression of *CSNK2A1* treated with endocrine therapy (top) or chemotherapy (bottom). HR—hazard ratio.

To identify major signaling events in regulation of quiescence entry and maintenance, we analyzed phosphorylation motifs that were upregulated in cells after 96 h of serum starvation (Figure 5A). One of the motifs whose phosphorylation was upregulated was pS-D-D/E-D/E, which is targeted by CK2 (Figure 5A). To test the hypothesis that CK2 substrates phosphorylation is upregulated after serum removal, we analyzed the cell lysates using a pCK2 substate antibody. This antibody recognizes the phosphorylated S/T-DXE motif. We first analyzed MDA-MB-231 as a cell line where we conducted the phosphoproteomic analysis. We found out that there was a significant increase in phosphorylation of the S/T-DXE motif after serum removal across various molecular weights, confirming our previous results (Figure 5B, left). We could see a gradual decrease in signal strength at some molecular weights which could indicate that as the cells go back to the cell cycle, CK2 activity is reduced to a basal level. Similar results were obtained in Hs578T and BT-549 cells; however, they were less pronounced than in MDA-MB-231 (Figure 5B, center and right). Because we saw increased CK2 activity in stressed conditions, we asked whether CK2 could be important for therapy response. Thus, we analyzed the TCGA dataset using the KMplot web interface [40]. We first looked at the expression of *Csnk2a1* mRNA in subtypes of breast cancer. We found out that, while in luminal A subtype the expression of *Csnk2a1* was not associated with worse patient survival (Figure 5C), in other subtypes (luminal B, Her2-enriched and Basal) the expression was significantly negatively correlated with patient survival. This association was the most significant in the basal subtype with HR = 2.38 (Figure 5C). Furthermore, we analyzed the association of *Csnk2a1* expression and response to therapy. We saw that there is no effect of *Csnk2a1* expression levels on the response of endocrine therapy (Figure 5D, top) standardly used in the Luminal A subtype [58]. On the contrary, there was a strong negative correlation between *Csnk2a1* expression and the outcome of chemotherapy (Figure 5D, bottom) that is standardly used for the basal subtype.

### 3.6. Inhibition of CK2 Has Noticeable Effects on Various Cellular Pathways

**Figure 6:**
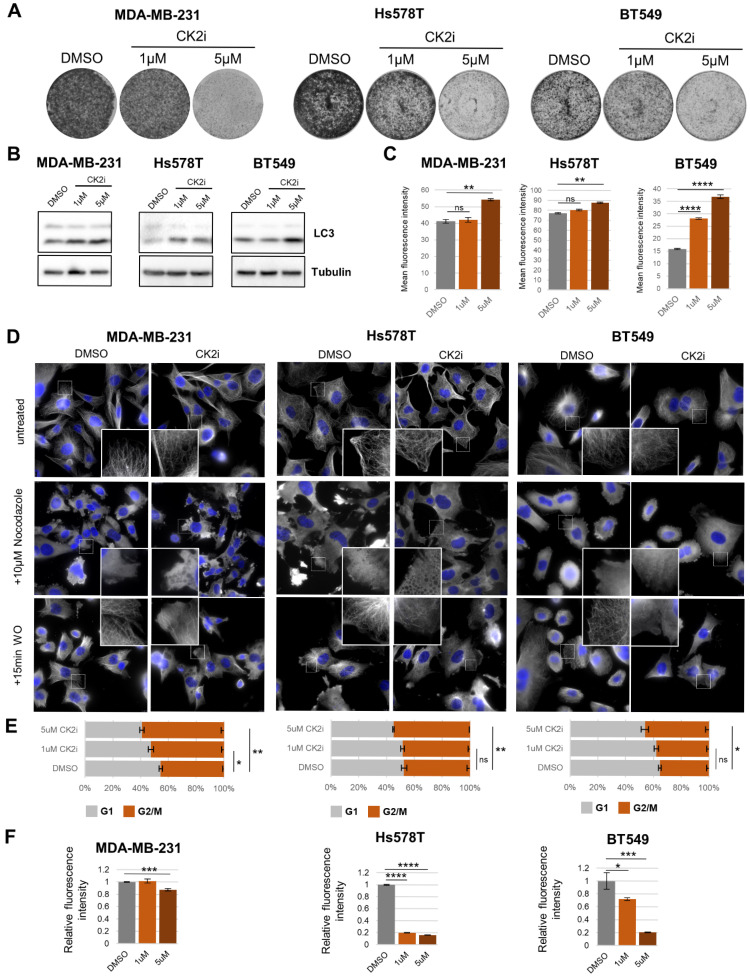
CK2 inhibition disrupts autophagy, microtubule dynamics, and protein synthesis. (**A**) Colony formation assay images for indicated TNBC cell lines treated with DMSO or indicated concentrations of CX-4945. (**B**) Expression of indicated proteins was analyzed using Western blot in indicated cell lines treated with DMSO or CX-4945. (**C**) Bar charts representing mean fluorescence intensity of cells treated with indicated concentrations of CX-4945 stained with Lysotracker^TM^ and analyzed by flow cytometry. ns—not significant, **, and **** represent *p*-values < 0.01, and <0.0001. (**D**) Representative images of indicated TNBC cell lines subjected to nocodazole washout experiment, pretreated with CX-4945 or DMSO as control. Blue—DAPI (nuclei), gray—αTubulin (microtubules). (**E**) Bar charts representing proportion of cells in indicated phases of cell cycle analyzed by flow cytometry. ns—not significant, * and ** represent *p*-values < 0.05, and <0.01. (**F**) Bar charts representing relative fluorescence intensity of cells treated with indicated concentrations of CX-4945 subjected to Click-IT HPG de novo proteosynthesis assay and analyzed by flow cytometry. *, ***, and **** represent *p*-values < 0.05, <0.001, and <0.0001.

To further investigate the role of CK2 in TNBC cells, we utilize the highly specific inhibitor CX-4945 (silmitasertib) [59]. First we tested the effect of CK2 inhibition on growth of TNBC cell lines using a colony formation assay. We saw that at low concentrations (1μM) there is a moderate decrease in proliferation in all three cell lines (Figure 6A), while increasing the concentration to 5μM exerted a strong growth inhibition effect (*p* < 0.01, *t*-test) (Figure 6A) consistent with previous results [60,61]. Furthermore, we wanted to validate the role of CK2 in the context of previously published results. To this aim, we analyzed the effect of CK2 inhibition on authophagy, which is one of the known pathways regulated by CK2 [62,63]. We treated the cells with CX-4945 and analyzed the authophagy induction by Western blot for LC3 isoforms. Our results show that CK2 inhibition stimulated an increased level of the smaller LC3 isoform in all three cell lines at 5 μM, and in MDA-MB-231 and Hs578T even at 1 μM (Figure 6B). We further confirmed our results from Western blot using LysoTracker (Invitrogen) staining followed by flow cytometry. LysoTracker is a fluorescent dye that labels acidic intracellular compartments, such as lysosomes and autolysosomes. In the context of autophagy, an increased LysoTracker signal reflects the accumulation of acidic vesicles associated with late stages of the autophagic process [64]. CK2 inhibition increased the size of the autophagosomes leading to an increased overall LysoTracker signal detected by flow cytometry in all three cell lines (Figure 6C). Moreover, CK2 has been implicated in regulation of microtubule dynamics [65,66]; thus, we wanted to validate that CK2 retains this function in our conditions as well. To this aim, we performed a nocodazole washout experiment to investigate the regrowth dynamics of microtubules after depolymerization. We compared untreated cells with cells that were pretreated with CX-4945 for 1 h and qualitatively analyzed the regrowth of microtubules 15 min after the nocodazole washout. The experiments showed that repolymerization of microbutules is significantly impaired in CX-4945-pretreated cells in comparison to the control. Importantly, the effect is consistent across all three analyzed cell lines (Figure 6D, bottom row, small image inserts). Additionally, we performed cell cycle analysis using flow cytometry. We revealed that CK2 inhibition leads to an increased proportion of G2/M phase cells, which indicates involvement of microtubules consistent with our previous results (Figure 6E). Taken together, we could draw the conclusion that there is a gradient of sensitivity to CK2 inhibition in the tested cell lines. Our results consistently indicate that the MDA-MB-231 cell line is the least sensitive while Hs578T and BT-549 are much more sensitive to CK2 inhibition. Finally, to tie the function of CK2 back to the results from our phosphoproteomic analysis, we analyzed two pathways that contained a high number of putative CK2 substrates based on the CK2 consensus sequence—proteosynthesis and microtubule dynamics. First we investigated the role of CK2 in sustaining proteosynthesis using Click-iT™ HPG Protein Synthesis Assay (ThermoFisher). We pretreated the cells using CX-4945 for 24 h and then incubated the cells with a L-homopropargylglycine (HPG) reagent to label nascent proteins for 2 h. Subsequent flow cytometry analysis revealed that CK2 inhibition leads to decreased nascent proteosynthesis in all three cell lines, but to a various degree (Figure 6F). In MDA-MB-231, we saw significant impairment of nascent proteosynthesis only in cells treated with 5μM CX-4945 (*p* < 0.001, *t*-test). In other two cell lines (Hs578T, BT-549), the effect of CK2 inhibition on proteosynthesis was much more profound even at 1 μM CX-4945 (BT-549: *p* < 0.05, Hs578T: *p* < 0.001, *t*-test), with the strongest effect seen in Hs578T (Figure 6F).

### 3.7. CK2 Supports Stress Survival by Suppressing DAPK3-Mediated Apoptosis in TNBC Cells

**Figure 7:**
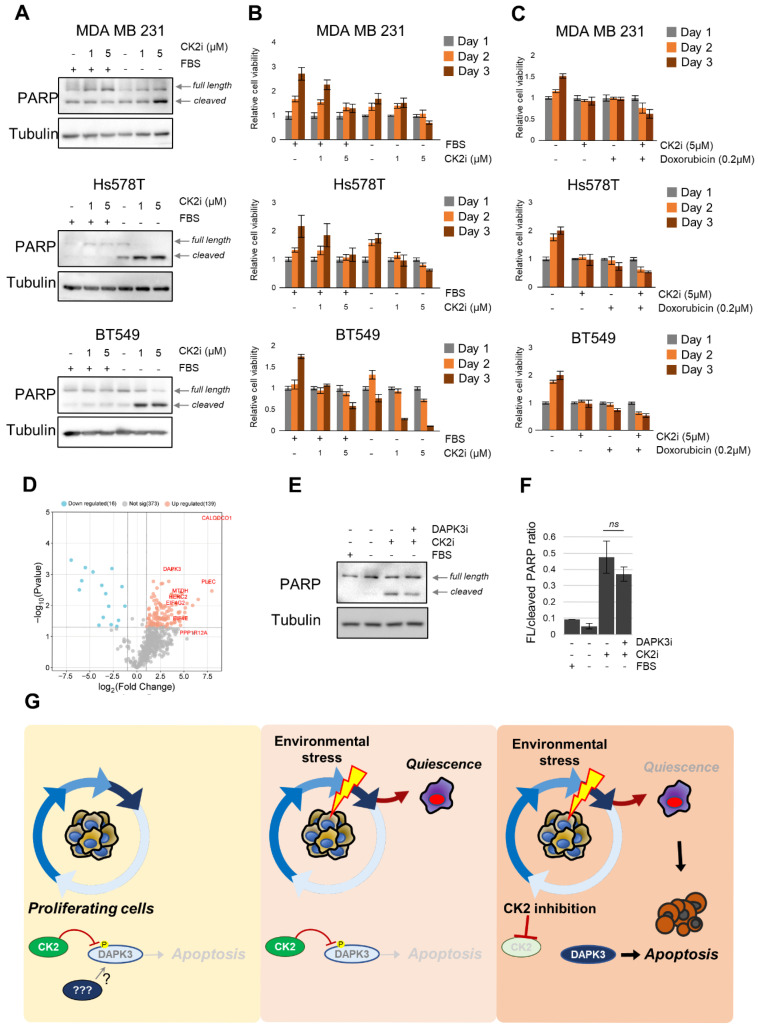
CK2 regulates DAPK3 to maintain survival under stress conditions. (**A**) Expression and cleavage of PARP1 was analyzed in MDA-MB-231, Hs578T and BT-549 under indicated conditions (+/− FBS, +/− CK2 inhibition) by Western blot. α-Tubulin was used as loading control. (**B**) TNBC cell lines (MDA-MB-231, Hs578T, BT-549) were treated with indicated concentrations of CX-4945 in serum containing as well as serum-free conditions and analyzed for cell viability using the MTT assay. Relative viability for each cell line relative to DMSO-treated cells at day 1 at respective condition is shown. (**C**) TNBC cell lines (MDA-MB-231, Hs578T, BT-549) were treated with indicated concentrations of CX-4945 and Doxorubicin and analyzed for cell viability using the MTT assay. Relative viability for each cell line relative to DMSO-treated cells at day 1 at respective condition is shown. (**D**) Volcano plot representing upregulated and downregulated proteins identified through phospho-CK2 substrate antibody pull-down after treatment of MDA-MB-231 with CX-4945. (**E**) Expression and cleavage of PARP1 was analyzed in MDA-MB-231 under indicated conditions (+/− FBS, +/− CK2 inhibition, +/− DAPK3 inhibition) by Western blot. α-Tubulin was used as loading control. (**F**) Bar chart representing quantification of PARP1 cleavage shown as ratio of full-length (FL) PARP1 to cleaved PARP1 from three independent experiments. ns—not significant. (**G**) Proposed model representing interaction of CK2 and DAPK3 in TNBC cells.

Finally, we wanted to understand the role of CK2 in response to stress. To this aim, we withdrew FBS to stimulate nutrient stress or treated the cells with doxorubicin to mimic anticancer therapy standardly used for TNBC treatment [67]. Simultaneously, we treated the cells with CX-4945. In a serum-starved condition, our experiments revealed that the viability of cells is compromised in serum-starving cells treated with CX-4945, as assessed by the cell survival assay as well as the PARP1 cleavage (Figure 7A,B). Serum removal or CK2 inhibition individually decreased the rate of proliferation. However, inhibition of CK2 in combination with serum removal had a more profound detrimental effect on cell viability and PARP1 cleavage (Figure 7A,B). When comparing individual cell lines, we see that MDA-MB-231 cells were the least sensitive to CK2 inhibition in combination with serum removal (Figure 7A,B, top row). Hs578T and BT-549 were much more sensitive to CK2 inhibition in serum-starved conditions and even 1 μM of CX-4945 resulted in strong PARP1 cleavage and cell viability decrease (Figure 7A,B, middle and bottom row). Additionally, we performed similar experiments with genotoxic stress mimicking anticancer therapy using doxorubicin. The results are aligned with our experiments using serum removal as a stress factor. Treatment of MDA-MB-231 and BT-549 cell lines with CX-4945 (5μM) potentiates the effect of doxorubicin even at a 0.2 μM concentration, where we saw a significant cytotoxic effect after 48 and 72 h (Figure 7C). In Hs578T cells, the co-treatment with CX-4945 (5μM) and doxorubicin (0.2 μM) led to a significant decrease in proliferation in comparison to CK2i or doxorubicin alone (Figure 7C). Furthermore, we also assessed the apoptosis induction using PARP1 cleavage in these conditions. In MDA-MB-231, the inhibition of CK2 potentiates the cytotoxic effect of doxorubicin where 0.5 μM doxorubicin leads to minimal PARP1 cleavage (2.8%) without CK2 inhibition. On the other hand, the same concentration of doxorubicin along with CK2 inhibition leads to substantial PARP1 cleavage (34.5%) (Figure S2A). In more sensitive cell lines Hs578T and BT-549, the combinatory treatment had a profound effect even at lower concentrations. Cotreatment of 0.2μM doxorubicin and CX-4945 resulted in significant PARP1 cleavage (Hs578T: 1.7% cleaved PARP1 in dox-only and 20.2% cleaved PARP1 in dox + CK2i; BT-549: 26.5% cleaved PARP1 in dox-only and 77.9% cleaved PARP1 in dox + CK2i) (Figure S2B,C). To further characterize CK2 dependencies in TNBC, we sought to identify downstream targets of CK2. To this end, we treated the MDA-MB-231 cells with CX-4945 and then performed pull-down using the pCK2 substrate antibody. Our analysis identified 528 proteins present in all six experiments (2 triplicates +/− CX-4945) out of which 139 have a significantly higher presence in non-treated pull-down in comparison to treated cells (Figure 7C). We have identified known CK2 substrates such as EIF4E, EIF4G2, EIF3D, MDC1, MYH10, CAPZA1 [68,69] among the enriched proteins in non-treated conditions, and there were multiple previously not described or validated substrates. Pathway analysis of putative substrates showed enrichment of CK2-regulated pathways such as translation regulation, RNA metabolism, stress response and autophagy (Figure S3A). One of the most enriched proteins in control samples was death-associated protein kinase 3 (DAPK3), which has been implicated in regulation of authophagy, apoptosis and cellular contractility [70,71,72]. Additionally, several of the DAPK3 interacting partners such as MYL9, PPP1R12A, CALM3 or LUZP1 were also enriched in our pull-down analysis (Figure S3B). Finally, in silico sequence and structure analysis confirmed the presence of putative CK2 phosphorylation consensus motif (T^112^EDE) located on the surface, and interestingly, mutation of this site impairs DAPK3’s kinase activity [73] (Figure S3C). Therefore, we decided to investigate functional association of CK2 and DAPK3. To this end, we stressed MDA-MB-231 cells by serum deprivation, treated the cells with CK2 (CX-4945) and DAPK3 (HS94) inhibitors and analyzed apoptotic signaling by PARP1 cleavage. As expected, inhibition of CK2 in serum-starved cells leads to a significant increase in PARP1 cleavage. Co-treatment with HS94 partially rescued the increased PARP1 cleavage (Figure 7F), indicating a functional interaction between CK2 and DAPK3. Taken together, we propose a model where, in normal growth conditions, pro-apoptotic function of DAPK3 is inhibited by CK2 and likely also other kinases through phosphorylation. When cells encounter adverse environmental conditions, the activity of pro-survival pathways is decreased while the activity of CK2 is sustained. This enables the inhibition of the pro-apoptotic function of DAPK3. Finally, inhibition of CK2 could lead to increased activity of DAPK3 and subsequent cell death (Figure 7G).

## 4. Discussion

The ability of cancer cells to enter the quiescent mode poses a major challenge for complete cancer eradication and is crucial for the emergence of minimal residual disease (MRD). These cells, which reside in a non-proliferative, dormant state, often evade conventional therapies that target rapidly dividing populations. As a result, they can remain undetected after initial treatment, contributing to MRD and posing a significant risk for relapse. Therefore, understanding the molecular mechanisms that maintain quiescence, allow cancer cells to survive long-term in such state and resist therapy is critical for developing strategies to eradicate MRD and achieve long-term remission.

Although the core circuits regulating mammalian cell quiescence such as CDKs, CKIs or Rb1 are well described [54,74] and are in place in cancer cells as well to a certain degree, targeting of these mechanisms is not feasible without affecting normal cells. Therefore, it is crucial to find upstream regulatory mechanisms specific to cancer cells; however, to date there is a limited number of large-scale proteomic analyses dynamically capturing the transition from proliferation to quiescence and back to the cell cycle. More importantly, quiescence is not a passive stage—it is actively maintained [75,76,77]. It has been shown that protein posttranslational modifications such as ubiquitination and phosphorylation are key events regulating the progression through the cell cycle. Therefore, we performed integrative proteomic and phosphoproteomic analysis of transition between proliferation and quiescence of cancer cells to capture the dynamics of protein phosphorylation and overall protein remodulation.

As expected, our analysis reveals that the proteome is significantly remodeled as cancer cells exit the cell cycle with the most pronounced downregulation seen in mitosis-promoting proteins such as CCNB1, DLGAP5 or AURKA. On the other hand, there is a significant increase in production of extracellular matrix (ECM) proteins and regulators such as FN1, THBS1 or PTX3. Increased production and stabilization of ECM is an adaptive mechanism in stress response and might be an inherent characteristic of quiescent niche [49]. Indeed, it has been shown that quiescence breast cancer cells upregulate the production of the extracellular matrix which is crucial for quiescence maintenance and survival of these cells [49]. ECM provides survival signaling mediated through integrins which supplements the lack of growth-factor stimulated survival signals [78]. Additionally, thicker layers of ECM provide shielding from environmental factors and, in the context of cancer biology, they protect quiescent cells from therapy and immune cell attacks [79,80]. The key role of ECM in quiescence regulation is further emphasized by strong degradation of FN1 or THBS1 at the exit of cells from quiescence. On the other hand, one of the key proteins for cell reactivation identified in our screen is HMGN2. HMGN2 is a nucleosome remodeling factor that has been implicated in the maintenance of the poised chromatin state in different systems [81]. This poised state is characteristic for quiescent cancer stem cells which stimulates plasticity [82]. Moreover, HMGN2 is important for efficient expression of early cell cycle regulatory genes and its mRNA is relatively stable during the cell cycle [83,84]. In line with our data, we could speculate that HMGN2 is expressed at a basal level in quiescent cells to control reversibility of the cell cycle exit. Upon mitogenic stimulation, the translation is increased, facilitating expression of early G1 genes such as Jun B, Jun D or FosL1 whose expression is significantly increased after 120 min of mitogenic stimulation in our conditions.

Our phosphoproteomic analysis also yields results consistent with the state of knowledge while also identifying a potential novel regulatory circuit in breast cancer cell quiescence. Specifically, looking at the regulation of the quiescent state, we have identified CK2 as potentially critical for the survival of quiescent cells. CK2 is a constitutively active serine/threonine kinase that was implicated in regulation of various cellular pathways through phosphorylation of its substrates [85]. Kinase motif analysis of our data identified phosphorylation of the CK2 consensus acidic sequence (S/T-D/E-D/E-D/E) to be increased in cells entering the quiescence. A more detailed analysis of known CK2 substrates shows increased phosphorylation of SCRIB, EDC4, CIC, SAP30, MAP1B, SP4 and STARD3NL at the transition to quiescence, indicating that CK2 activity might be either directly regulating entry into the quiescence or that it is important for survival of quiescent cells. We confirmed increased phosphorylation of CK2 substrates in two different TNBC cell lines and to a certain degree also in the third one—BT-549. Differences in the third cell line might stem from the lack of the Rb1 protein, which is one of the key core regulators of cell quiescence [86,87]. Interestingly, when we look at the association of CK2 expression and response to therapy, we see that patients with low expression of *CSNK2A1*—gene coding the alpha subunit of CK2—are responding very well to cytotoxic therapy, while patients with higher CSNK2A1 expression have significantly worse clinical outcomes. Since cytotoxic therapy is one of the stimuli driving quiescence as a survival mechanism [5,13], it further strengthens our hypothesis that CK2 is important for cancer cell quiescence and minimal residual disease.

Finally, we wanted to test whether inhibition of CK2 would lead to lower survival of QCCs. To this aim, we stressed the cells by removal of the serum to stimulate nutrient depletion-induced quiescence as well as by adding doxorubicin to mimic the anticancer therapy-induced quiescence. Inhibition of CK2 in these stressed conditions led to significant decrease in viability of all three tested cell lines, which indicates that CK2 activity is indeed important for survival of cells upon stress. It has been previously shown that CK2 is activated upon various stress conditions in a p38-dependent manner [88,89]. Increased activity of CK2 then leads to phosphorylation of its downstream targets that are important for survival upon stress, such as NRF2, HSP90 or JNK1 [25,90,91]. To further delineate the pathway downstream of CK2 in our model system, we performed a pull-down experiment using a pCK2 substrate antibody and identified the proteins differentially phosphorylated in cells treated with CX-4945 with vehicle-treated cells. Besides known CK2 substrates such as EIF4E, EIF4G2, MYH10 and CAPZA1 [68,69], we identified death-associated protein kinase 3 (DAPK3) as a potential CK2 substrate, being one of the proteins with the most significant downregulated phosphorylation after CK2 inhibition. DAPK3 has been initially identified as a pro-apoptotic kinase, integrating signals from various stress pathways and promoting apoptosis and autophagy [70,71,91]. DAPK3 sequence analysis reveals that it contains one potential CK2-phosphosite—Threonine 112—followed by a stretch of acidic amino acids E-D-E, which corresponds to the CK2 phosphorylation consensus [92]. Interestingly, cancer-associated missense mutation of this site was associated with decreased activity of DAPK3, suggesting its role as a tumor suppressor kinase [73]. We hypothesized that CK2-mediated phosphorylation of T112 could lead to the downregulation of DAPK3 activity or altered localization which might result in inhibition of apoptosis mediated by the lack of extracellular pro-survival signaling. To test the hypothesis, we treated serum-starved cells with CK2i as well as DAPK3i and analyzed apoptotic signaling through PAPR cleavage. Our data show that inhibition of DAPK3 leads to partial rescue of PARP1 cleavage in response to serum starvation and CK2 inhibition. The results are in line with published literature on the pro-apoptotic role of DAPK3 [93,94], as well as the importance of T112 for DAPK3 functionality [73]. Additionally, our model is supported by another high-throughput study that shows that CK2-targeted phosphosites are significantly enriched in gefitinib-resistant PC9 cells compared to parental cells, and one of the sites phosphorylated by CK2 was T112 on DAPK3 [93].

While our integrative proteomic and phosphoproteomic approach provides critical insights into the signaling dynamics of QCCs, we acknowledge several limitations. Our analyses were conducted in vitro in TNBC cell lines. Although we validated several key findings across three independent models and in-patient datasets, future studies involving patient-derived organoids or in vivo models will be necessary to confirm the physiological relevance of CK2-dependent signaling in quiescence and minimal residual disease (MRD). Furthermore, while CK2 was implicated as an important survival kinase in QCCs, the exact mechanism by which CK2 regulates downstream effectors such as DAPK3 remains to be fully elucidated. Phosphorylation of DAPK3 at T112 appears functionally relevant, but additional studies including site-directed mutagenesis, phospho-deficient or phospho-mimetic constructs and rescue experiments are needed to validate its regulatory role.

## 5. Conclusions

In summary, our study provides the first comprehensive phosphoproteomic characterization of TNBC cells transitioning into and out of quiescence and supports the role of CK2 as an important regulator of survival in the quiescent state. We demonstrate that CK2 activity is enhanced during cellular stress, supports survival signaling, and suppresses pro-apoptotic pathways through modulation of targets such as DAPK3. These findings suggest that CK2 enables QCCs to withstand both nutrient deprivation and cytotoxic therapy, contributing to minimal residual disease and relapse. Targeting CK2 in combination with conventional therapies may represent a promising strategy to eliminate therapy-resistant quiescent cancer cell populations and improve long-term treatment outcomes in aggressive breast cancer subtypes. Nonetheless, further in vivo and mechanistic studies are required to fully validate CK2’s role in quiescence regulation and therapeutic resistance.

## Data Availability

The datasets generated during and/or analyzed during the current data have been deposited to the ProteomeXchange Consortium via the PRIDE [95] partner repository with the dataset identifier PXD071322.

## Supplementary Materials

The following supporting information can be downloaded at: https://www.mdpi.com/article/10.3390/cancers18091449/s1, Figure S1: Validation of quiescence induction by Western blot; Figure S2: CK2 inhibition potentiates doxorubicin-induced apoptosis in TNBC cells; Figure S3: Functional and structural analysis of DAPK3; File S1: Differential site-specific phosphorylation during serum starvation-induced quiescence; Table S1: Antibodies used in the study.
