# Supplementary material for: Dynamic Phosphoproteomic Profiling Identifies Casein Kinase 2 as a Critical Survival Kinase in Quiescent Breast Cancer Cells and a Potential Therapeutic Target for Minimal Residual Disease
Source: Cancers (Basel). 2026 Apr 30;18(9):1449. doi: 10.3390/cancers18091449 (PMC13163072; doi:10.3390/cancers18091449)
Supplement: Supplementary file 1 [file cancers-18-01449-s001.zip › Table S1_revised.pdf]

Table S1. Antibodies used in the study

Antibody	Source	Catalogue Number
Anti-p27 [Kip1]	BD Biosciences	Cat# 554069
Anti-p4EBP1 (Thr70)	Cell Signaling Technology	Cat# 9455
Anti-4EBP1 (53H11)	Cell Signaling Technology	Cat# 9644
Anti-pRb (S807/811) (D20B12)	Cell Signaling Technology	Cat# 8516
Anti-Rb1 (4H1)	Cell Signaling Technology	Cat# 9309
Anti-pCK2 substrate [(pS/pT)DXE]	Cell Signaling Technology	Cat# 8738
Anti-PARP	Cell Signaling Technology	Cat# 9542
Anti-E2F8	SantaCruz Biotechnology	Cat# sc-514064
Anti-DLGAP5	SantaCruz Biotechnology	Cat# sc-377004
Anti-CK2 IIα	SantaCruz Biotechnology	Cat# sc-373894
Anti-α tubulin (DM1A)	SantaCruz Biotechnology	Cat# sc-32293
Anti-LC3 (G-8)	SantaCruz Biotechnology	Cat# sc-376404
Anti-RPS6 (C-8)	SantaCruz Biotechnology	Cat# sc-74459
Anti-pRPS6 (50.Ser 235/236)	SantaCruz Biotechnology	Cat# sc-293144
