# Supplementary material for: Dynamic Phosphoproteomic Profiling Identifies Casein Kinase 2 as a Critical Survival Kinase in Quiescent Breast Cancer Cells and a Potential Therapeutic Target for Minimal Residual Disease
Source: Cancers (Basel). 2026 Apr 30;18(9):1449. doi: 10.3390/cancers18091449 (PMC13163072; doi:10.3390/cancers18091449)
Supplement: Supplementary file 1 [file cancers-18-01449-s001.zip › Supplementary Figures.pdf]

Figure S1:

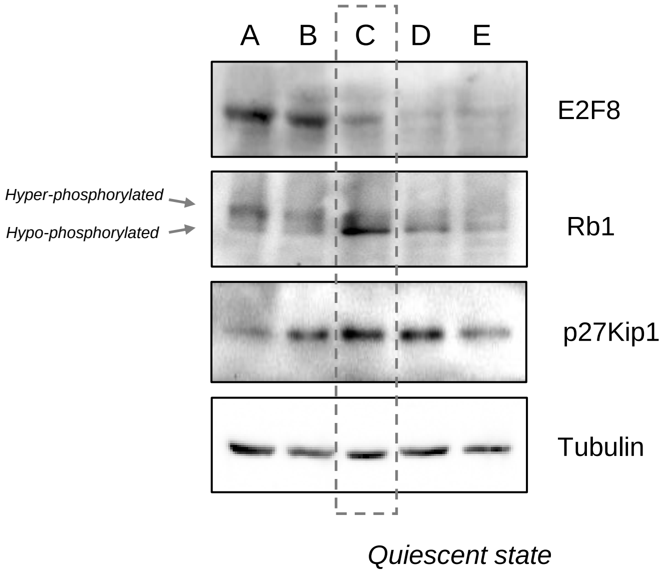

Figure S1. Validation of quiescence induction by western blot. Expression of indicated proteins was analyzed in MDA-MB-231 in various timepoints corresponding to the transition between quiescence and proliferation. Timepoints were as follows: *A* – continuously growing cells; *B* – serum starved cells (48 hrs); *C* – serum starved cells (96 hrs); *D* - serum reactivated cells (+20 min); *E* - serum reactivated cells (+120 min). α-tubulin was used as loading control.

Figure S2:

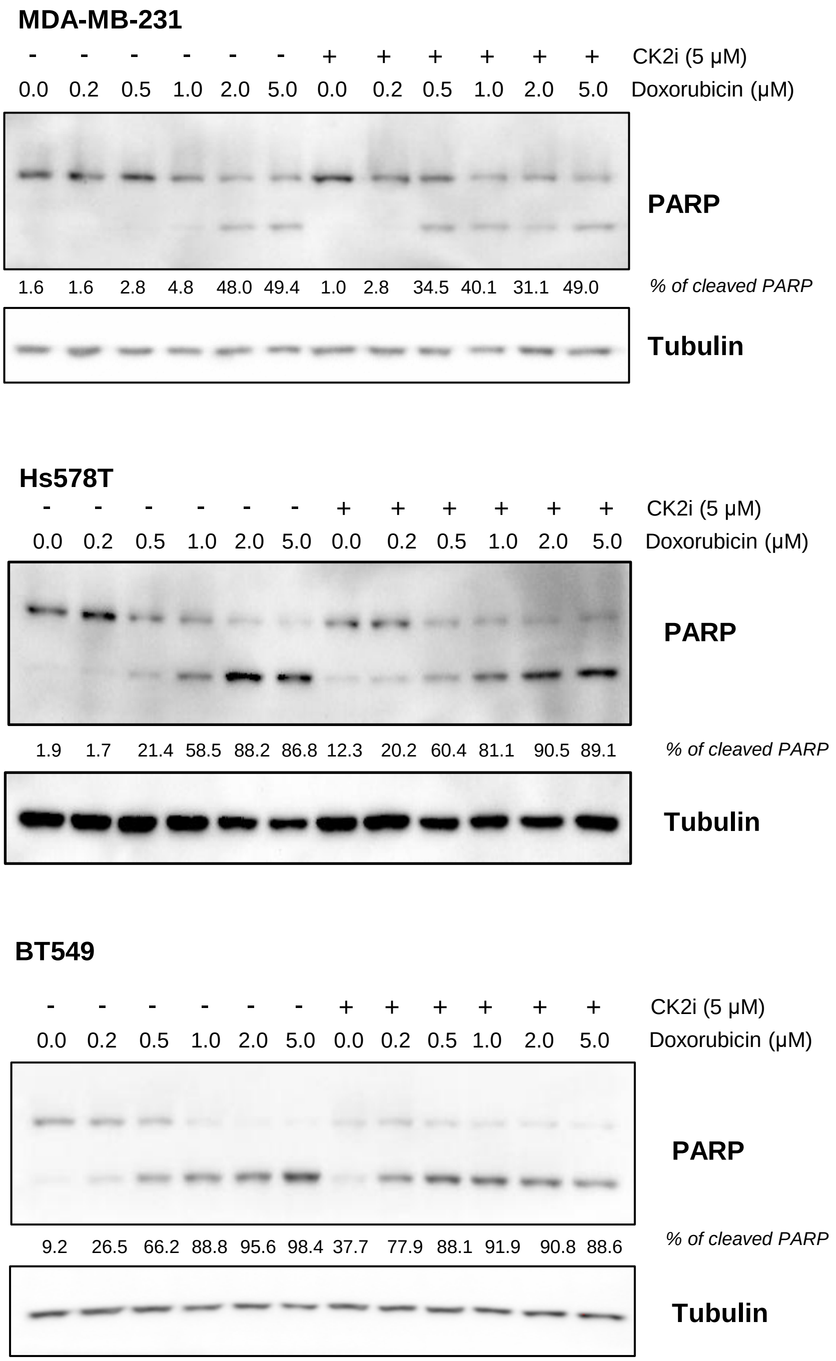

Figure S2. CK2 inhibition potentiates doxorubicin-induced apoptosis in TNBC cells. Expression and cleavage of PARP was analyzed in MDA-MB-231, Hs578T and BT549 under indicated conditions (+/- CK2 inhibition, +/- Doxorubicin) by western blot. α-Tubulin was used as loading control. Numbers indicate percentage of cleaved PARP in corresponding conditions.

Figure S3:

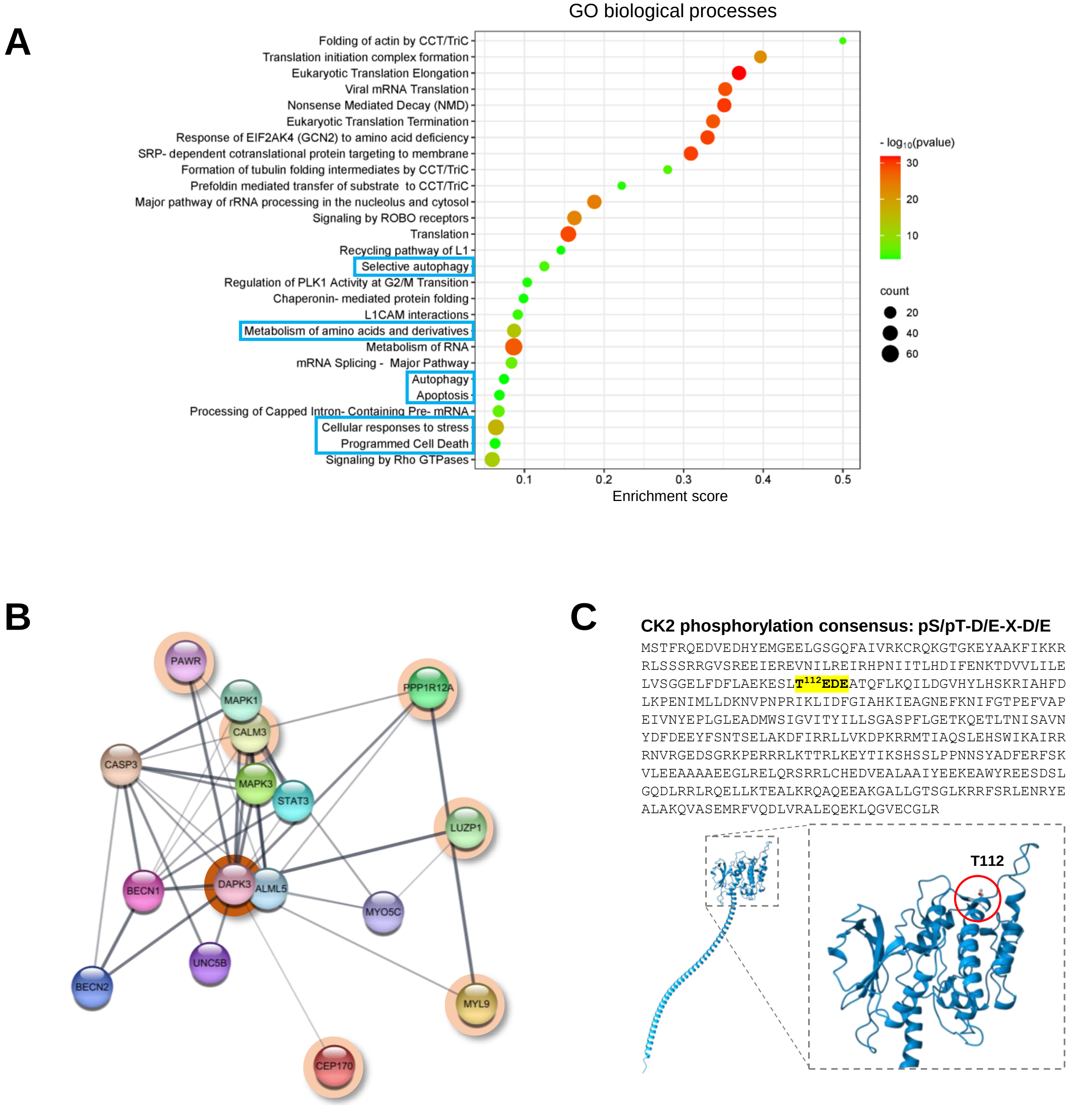

Figure S3. Functional and structural analysis of DAPK3 (A) GO Biological Processes enrichment analyses showing enriched pathways identified through phospho-CK2 substrate pulldown analysis. Color indicates false discovery rate (FDR), circle size indicates number of identified hits in the pathway, X-axis shows enrichment score. **(B)** Network analysis of DAPK3 visualized using String database (https://string-db.org/) with highlighted proteins identified in the pulldown. **(C)** Primary amino acid sequence of DAPK3 with threonine 112 highlighted in red. Threonine 112 resides within a CK2 consensus motif (T^{112}EDE), positioned on a solvent-accessible surface of the kinase domain.
